# INFRAFRONTIER quality principles in systemic phenotyping

**DOI:** 10.1007/s00335-021-09892-2

**Published:** 2021-07-30

**Authors:** Hilke Ehlich, Heather L. Cater, Ann M. Flenniken, Isabelle Goncalves Da Cruz, Anne-Marie Mura, Vasileios Ntafis, Michael Raess, Mohammed Selloum, Claudia Stoeger, Sarka Suchanova, Reetta Vuolteenaho, Steve D. M. Brown, Yann Hérault, Reetta Hinttala, Martin Hrabě de Angelis, George Kollias, Dimitris L. Kontoyiannis, Bernard Malissen, Colin McKerlie, Radislav Sedláček, Sara E. Wells, Ana Zarubica, Jan Rozman, Tania Sorg

**Affiliations:** 1 INFRAFRONTIER GmbH, Neuherberg, Germany; 2 MRC Harwell Institute, Mary Lyon Centre, Harwell Campus, Oxfordshire, UK; 3 Lunenfeld-Tanenbaum Research Institute, Sinai Health, Toronto, ON Canada; 4 The Centre for Phenogenomics, Toronto, ON Canada; 5 Universite de Strasbourg, CNRS, INSERM, CELPHEDIA, PHENOMIN, Institut Clinique de La Souris, Illkirch, France; 6 Centre d’Immunophénomique (CIPHE), Aix Marseille Université, INSERM, CNRS, CELPHEDIA, PHENOMIN, Marseille, France; 7 Biomedical Sciences Research Center “Alexander Fleming”, Athens, Greece; 8 Helmholtz Zentrum München, Institute of Experimental Genetics, German Mouse Clinic, Neuherberg, Germany; 9 Czech Centre for Phenogenomics, Institute of Molecular Genetics of the Czech Academy of Sciences, Vestec, Czech Republic; 10 University of Oulu, Biocenter Oulu, Transgenic and Tissue Phenotyping Core Facility, Oulu, Finland; 11 MRC Harwell Institute, Mammalian Genetics Unit, Medical Research Council, Harwell Campus, Oxfordshire, UK; 12 University of Oulu and Oulu University Hospital, PEDEGO Research Unit and Medical Research Center Oulu, Oulu, Finland; 13 German Center for Diabetes Research (DZD), Neuherberg, Germany; 14 Technische Universität München, Chair of Experimental Genetics, TUM School of Life Sciences, Freising, Germany; 15 The Hospital for Sick Children, Toronto, ON Canada; 16 The Faculty of Medicine, University of Toronto, Toronto, ON Canada

## Abstract

Improving reproducibility and replicability in preclinical research is a widely discussed and pertinent topic, especially regarding ethical responsibility in animal research. INFRAFRONTIER, the European Research Infrastructure for the generation, phenotyping, archiving, and distribution of model mammalian genomes, is addressing this issue by developing internal quality principles for its different service areas, that provides a quality framework for its operational activities. This article introduces the *INFRAFRONTIER Quality Principles in Systemic Phenotyping* of genetically altered mouse models. A total of 11 key principles are included, ranging from general requirements for compliance with guidelines on animal testing, to the need for well-trained personnel and more specific standards such as the exchange of reference lines. Recently established requirements such as the provision of FAIR (Findable, Accessible, Interoperable, Reusable) data are also addressed. For each quality principle, we have outlined the specific context, requirements, further recommendations, and key references.

**Supplementary Information:**

The online version contains supplementary material available at 10.1007/s00335-021-09892-2.

## Introduction

Low reproducibility in preclinical research is a widely-debated topic. The consequences of irreproducibility are manifold, the most obvious being reduced efficiency in the development of new treatments, the cost in animal lives, the waste of billions of US$ per year in the USA alone (Freedman et al. 2017) as well as the loss of trust in science.

Various efforts have been undertaken to increase reproducibility in animal research. Guidelines for experimental design and reporting have been formulated, e.g. PREPARE (Smith et al. 2018) and ARRIVE guidelines (Kilkenny et al. 2010, Percie du Sert 2020a, b), and funding bodies are actively fostering the sharing of data which is open and FAIR (Findable, Accessible, Interoperable and Reusable; Wilkinson et al. 2016) (European Open Science Cloud in the EU, Science Data Commons in the US).

In addition, large research consortia, such as the International Mouse Phenotyping Consortium (IMPC), which aim to phenotype knockouts for all protein-coding genes in the mouse genome, are driving improvements through standardisation of procedures and sharing of data.

INFRAFRONTIER is the European Research Infrastructure for the generation, phenotyping, archiving and distribution of model mammalian genomes. INFRAFRONTIER provides centralised access to mouse and rat models, data, and scientific platforms and services to study the functional role of the genome in human health and disease. The core services comprise model generation, archiving and distribution of mouse mutant lines by the European Mouse Mutant Archive (EMMA), and systemic phenotyping of mouse mutants in the participating mouse clinics. Systemic phenotyping, as used in the INFRAFRONTIER context, means the systematic analysis of mutant mice through a standardised phenotyping pipeline across a range of biological systems to infer gene function.

The INFRAFRONTIER consortium is currently formed by 29 partners across 14 European countries and Canada, with most of the mouse clinics also being members of the IMPC network. Accordingly, its contribution to enhancing reproducibility in biomedical research is characterised by providing centralised open access to high-quality scientific data, biomedical services and resources, and through the sharing of knowledge. As a large distributed research infrastructure, it is the mission of INFRAFRONTIER to deliver reliable services and reproducible data while advancing animal research in accordance with the 3Rs (Replacement, Reduction, Refinement).

## INFRAFRONTIER quality principles in systemic phenotyping

INFRAFRONTIER relies on the development of internal quality principles to provide a quality framework for its operational activities and to guide continuous improvement in response to the needs of users and stakeholders. Incorporating both existing operational standards and common concepts in Quality Management (QM), these principles define internal key quality standards for each INFRAFRONTIER service. In addition, current developments and insights towards enhanced research reproducibility are addressed. These include providing transparent, FAIR and open data, following the ARRIVE and PREPARE guidelines, and using the most advanced experimental approaches. Since INFRAFRONTIER is a consortium of many research institutions across different countries, the Quality Principles recognise that requirements at each of the national nodes may differ and that different strategies may be chosen for the application of the defined standards.

This article provides a complete overview of the *INFRAFRONTIER Quality Principles in Systemic Phenotyping*. These principles were established by the INFRAFRONTIER Quality Management Network Group, an internal INFRAFRONTIER group composed of representatives of the INFRAFRONTIER partner institutions, who are dedicated to the exchange of current QM topics. The group identified the following 11 principles as crucial for providing reliable, high-quality phenotyping services:

1. We strictly comply to national and international legislation on ethics and animal welfare
2. We promote and apply the 3Rs (Replacement, Reduction, Refinement)
3. We apply good experimental practice
4. We apply Standard Operating Procedures
5. We ensure that our procedures are carried out by competent and well-trained personnel
6. We apply reference ranges where feasible
7. We exchange and analyse reference lines or reference samples where feasible
8. We use appropriate statistical analyses that are fit for purpose
9. We report metadata
10. We advise that the data that we provide is FAIR (Findable, Accessible, Interoperable, Reusable)
11. We maintain and extend the mechanisms (working groups, training, exchange of experience) to constantly improve our data quality (Plan-Do-Check-Act)

In addition, the partners elaborated on context, specific requirements, recommendations, and supporting references sections for each of the 11 principles.

In this respect, the context sections are meant to “set the scene”. They provide information about the underlying background of the principles, for example specific conditions and circumstances which need to be considered. Thus, they point out the relevance of the specific principles for INFRAFRONTIER.

The requirement sections list mandatory points, such as procedures which should be implemented at the nodes to fulfil the needs of the relevant principle.

Additionally, the recommendation section of each principle proposes further procedures and measures which will improve adherence to the principles but are not mandatory.

Finally, the references list relevant regulations and publications, as well as links to webpages which are referred to in the other sections or provide useful information.

## Conclusion

By defining crucial quality criteria, the INFRAFRONTIER quality principles establish the basis for reproducible and reliable research results. They provide the participating partners with a sound framework for their operational activities in systemic phenotyping while at the same time providing leeway for the implementation of and adherence to national and local requirements.

## Supplementary Information

Below is the link to the electronic supplementary material.

Supplementary file1 (PDF 438 kb)
