# Supplementary material for: INFRAFRONTIER quality principles in systemic phenotyping
Source: Mamm Genome. 2021 Jul 30;33(1):120–2. doi: 10.1007/s00335-021-09892-2 (PMC8913563; doi:10.1007/s00335-021-09892-2)
Supplement: Supplementary file 1 — Supplementary file1 (PDF 438 kb) [file 335_2021_9892_MOESM1_ESM.pdf]

INFRAFRONTIER Quality Principle (1) in Systemic Phenotyping

**(1) We strictly comply to national and international legislation on ethics and animal welfare**
**Context:**<br>● INFRAFRONTIER Mission: Providing unique scientific resources, services, and expertise to advance the understanding, prevention, and treatment of human diseases using rodent models<br>● We apply the DIRECTIVE 2010/63/EU including its national transpositions (or as applicable the respective Canadian and UK legislations) and other relevant Animal Health and Welfare policies
**Requirements:**<br>● Site specific transposition and application of relevant international and national regulations and requirements (instructions etc.)<br>● Ethical review process by competent authorities, both internal and external<br>○ acceptability of research projects<br>○ follow progress of research projects<br>○ perform a retrospective review<br>○ review animal care and accommodation standards<br>○ evaluate the "cost/benefit" balance of research projects<br>○ verify compliance of research projects with legal requirements for replacement, reduction, and refinement<br>● Animal welfare body (internal and external)<br>○ advise staff and keep them up-to-date on animal welfare regulations and the 3Rs<br>○ ensure implementation of the research projects remains compliant, and outcomes are tracked
**Recommendations:**<br>● Follow the recommendations of the European Expert Groups:<br>https://ec.europa.eu/environment/chemicals/lab_animals/index_en.htm
**References:**<br>● DIRECTIVE 2010/63/EU (EU Parliament and Council 2010) and its applicable national transitions<br>● COMMISSION IMPLEMENTING DECISION (EU) 2020/569 (European Commission 2020)<br>● Guide to Care and Use of Experimental Animals. Vol 1, 2nd edition (CCAC 2020)<br>● CCAC Guidelines: Mice (CCAC 2019)<br>● The Animals (Scientific Procedures) Act 1986 Amendment Regulations 2012 (UK Statutory Instruments 2012)<br>● Working document on genetically altered animals (European Commission Expert Working Group 2013)<br>● Working document on a severity assessment framework (European Commission Expert Working Group 2012)<br>● Guide for the Care and Use of Laboratory Animals: Eighth Edition (National Research Council 2011)

INFRAFRONTIER Quality Principle (2) in Systemic Phenotyping

(2) We promote and apply the 3Rs (Replacement, Reduction, Refinement)

Context:

- Characterisation of genetically modified rodent models for human diseases has become a key tool in basic and biomedical research to understand molecular mechanisms of human disorders and for the development of new therapies
- The laboratory mouse shares over 90% of its genome with humans, as well as similar physiology and anatomy for the majority of organs and organ systems
- An extensive toolkit for the manipulation of the mouse genome exists including use of mouse Embryonic Stem cells and genome editing techniques such as CRISPR/Cas9 to create rodent models to investigate gene function and enhance our understanding of the genetic basis of human diseases
- INFRAFRONTIER is using these scientifically valuable rodent models to conduct comprehensive phenotyping pipelines, providing a wide breadth of clinical information per experimental animal to the scientific community, and thereby minimising overall animal usage
- Replacement, Reduction, Refinement of animal usage (3Rs concept) provides INFRAFRONTIER with a solid framework to continually rethink and optimise its experimental approaches in systemic phenotyping to improve the quality and reliability of research outputs
- According to the current guideline (EMA/CHMP/CVMP/JEG-3Rs/450091/2012, EMA 2016) the 3Rs are:
  - *Replacement*: testing approaches that avoid or replace the use of live animals in an experiment. Replacement could include the use of established animal and human cell lines or tissues, mathematical/computer models or physicochemical methods. In addition, according to NC3Rs (NC3Rs 2021, https://www.nc3rs.org.uk/the-3rs) partial replacement is the use of animals that, based on current scientific understanding, are not considered capable of experiencing suffering, such as Drosophila and nematode worms.
  - *Reduction*: approaches that minimise the number of animals used per experiment or study, either by enabling researchers to obtain comparable levels of information from fewer animals, or to obtain more information from the same number of animals, thereby avoiding further animal use. Examples include improved experimental design and statistical analysis, combination of studies, international harmonisation of testing requirements to avoid duplicate testing and the use of technologies, such as imaging, to enable longitudinal studies in the same animals.
  - *Refinement*: approaches that minimise the pain, suffering, distress or lasting harm that may be experienced by the animals. Refinement applies to all aspects of animal use, from the housing and husbandry to the scientific procedures performed. An example of refinement is the use of appropriate anaesthetics and analgesics.

Requirements:

- According to Directive 2010/63/EU on the protection of animals used for scientific purposes (EU Parliament and Council 2010), Article 4:
  - ensure that, wherever possible, a scientifically satisfactory method or testing strategy that does not use live animals is applied
  - ensure that the number of animals used in research projects is reduced to a minimum without compromising the objectives of the projects
  - ensure refinement of breeding, accommodation and care, and of methods used in procedures, to reduce or eliminate any pain, suffering, distress or lasting harm to the animals

- Reduction:
  - conduct extensive literature research during experimental planning to avoid any duplication of experiments
  - drafting of an experimental protocol before any experimentation
  - use of statistics in the design of the experimental protocol for *a priori* estimation of the necessary and sufficient number of animals
  - sharing of animals between research projects wherever possible
  - use of animals that are homogeneous in terms of biological characteristics and health status
  - ensure maximal exploitation of the data obtained during an experiment
- Refinement:
  - reduce, eliminate, or relieve discomfort, pain, distress or anxiety experienced by animals
  - carefully choose the animal model used, considering the current state of knowledge
  - improve the conditions of transport, breeding, and accommodation (e.g. care, enrichment of the environment)
  - carefully plan the protocol to avoid disruptions that may occur in the future
  - ensure that the objective of the research project complies with the established humane endpoints
  - utilise non-invasive procedures whenever possible
  - use anaesthetics and analgesics in accordance with veterinary advice and regulatory requirements
  - perform appropriate and humane killing methods
- Replacement:
  - replace animal models with *in vitro* or *in silico* models whenever appropriate
  - publish phenotyping data to avoid unnecessary repetition of animal studies
- Verify compliance to 3Rs requirements in ethical review processes

Recommendations:

- Constant technology development for the implementation of the 3Rs in experimental procedures
- Exchange of knowledge of 3R related developments between INFRAFRONTIER partners
- Apply the PREPARE Guidelines (Smith et al 2018) when designing phenotyping experiments
- Reporting according to ARRIVE Guidelines (Kilkenny et al 2010, Percie du Sert 2020a, Percie du Sert 2020b)

References:

- DIRECTIVE 2010/63/EU (EU Parliament and Council 2010) and its applicable national transitions
- National Competent Authorities for the implementation of Directive 2010/63/EU (European Commission Expert Working Group 2014)
- Guide to Care and Use of Experimental Animals. Vol 1, 2nd edition (CCAC 2020)
- CCAC Guidelines: Mice (CCAC 2019)
- Guideline on regulatory acceptance of 3Rs testing approaches EMA/CHMP/CVMP/JEG-3Rs/450091/2012 (EMA 2016)
- Dataset on alternative methods to animal experimentation DB-ALM (European Commission, Joint Research Centre 2019)
- PREPARE guidelines (Smith et al 2018)
- ARRIVE guidelines (Kilkenny et al 2010, Percie du Sert 2020a, Percie du Sert 2020b)

- NC3Rs website (NC3Rs 2021): 3Rs (https://www.nc3rs.org.uk/the-3rs), study design tool (https://www.nc3rs.org.uk/experimental-design-assistant-eda)

INFRAFRONTIER Quality Principle (3) in Systemic Phenotyping

(3) We apply good experimental practice

Context:

- Standardisation and harmonisation of practice and tests (within a phenotyping facility and across INFRAFRONTIER mouse clinics in Europe)
- Tests validated by using strain comparison or pharmacological validation whenever possible
- Controlled housing conditions / environment
- Define the most appropriate study design

Requirements:

- *A priori* power analysis (minimum number of animals required)
- Proper age-matched controls on the same genetic background (ideally littermates)
- Justify the genetic background: appropriate or not for a given test?
- Be aware about the health status and potential interference with the phenotyping assays
- Justify the housing and husbandry conditions (e.g. number of mice per cage)
- Justify the operational conditions (e.g. fasting time & duration, which anaesthesia, route of blood collection)
- Follow welfare considerations and regulations
- Ethical submission and validation of the project
- Apply the 3Rs

Recommendations:

- Two sexes are recommended
- If only one sex, then justify why
- Apply research rigour measures for bias reduction (e.g. randomisation, blinding) where feasible
- Apply statistics consultation
- Analyse each biological function by using different and complementary assays
- Obtain the maximum amount of information possible from a single test
- Apply tests validated previously by strain comparison or pharmacological validation whenever possible
- Get advice from the veterinarian, the ethical committee, the animal welfare structure in your organisation
- Design test sequence (e.g. phenotyping pipeline) from less to more invasive

References:

- Handbook: Quality practices in basic biomedical research (WHO TDR 2006)
- PREPARE guidelines (Smith et al 2018)
- ARRIVE guidelines (Kilkenny et al 2010, Percie du Sert 2020a, Percie du Sert 2020b)
- Meehan et al 2017
- NIH website (NIH 2021): Rigor and reproducibility (https://www.nih.gov/research-training/rigor-reproducibility)
- NC3Rs website (NC3Rs 2021): 3Rs (https://www.nc3rs.org.uk/the-3rs), study design tool (https://www.nc3rs.org.uk/experimental-design-assistant-eda)

INFRAFRONTIER Quality Principle (4) in Systemic Phenotyping

(4) We apply Standard Operating Procedures

Context:

- Key principle: to reach repeatability, reproducibility, and robustness at each step of the experiment (e.g. performing the assay, collecting data, analysing data), ensure transparency as well as management and transfer of knowledge
- Standard Operating Procedures (SOPs)
  - provide specifications and step-by-step instructions for (complex) routine operations / activities
  - regulate how phenotyping procedures are physically carried out as well as reproducible data handling and organization
  - provide means to minimise errors and improve uniformity of performance and quality output
- IMPC: A centralised database of consensus IMPC standard operating procedures (SOPs), IMPReSS, ensures that all phenotyping data and metadata are collected in a reproducible and standardised format (Meehan et al 2017)

Requirements:

- Implement and maintain SOPs for phenotyping tests and quality-related activities (e.g. data management, data analysis, animal welfare, training of staff)
- Define
  - responsibilities, operational methods, parameters, and metadata to be measured, recorded, evaluated, and reported, including quality control steps
  - controlled equipment and resources / consumables
- Periodic SOP review and SOP management (disseminate/train users on the latest SOP version, withdraw outdated versions)

Recommendations:

- Standardisation of procedures or phenotyping pipelines where appropriate (e.g. IMPC)
- Evaluate repeatability and reproducibility of assays (e.g. use one versus several batches of mice; inter-operator reproducibility)
- Report any deviations and assess them as appropriate

**References:**<br>● Handbook: Quality practices in basic biomedical research (WHO TDR 2006). Chapter 4.3.2 Standard operating procedures<br>● Meehan et al 2017<br>● IMPReSS website (IMPReSS 2021)

INFRAFRONTIER Quality Principle (5) in Systemic Phenotyping

**(5) We ensure that our procedures are carried out by competent and well-trained personnel**
**Context:**<br>● Each activity requires adequate qualification and training of involved personnel<br>● Training is essential to ensure that procedures are carried out in a reproducible way<br>● On-site training is complemented by INFRAFRONTIER training workshops and exchange of experience<br>● Application of / compliance to regulatory training requirements (e.g. European Directive 2010/63 and its applicable national transitions)<br>● Recording of personnel competencies is regulated by the QM systems applied in the different INFRAFRONTIER centres
**Requirements:**<br>● Definition of job requirements and responsibilities for each level of personnel<br>● Qualification of all personnel should be defined in education and training. Record qualifications in up-to-date training cards/plans to keep track of individual training status and demands<br>● All personnel who handle animals must be suitably qualified and undergo training to maintain and update their skills and their awareness of the 3Rs. Continual training should be provided in the form of regular on-site training, carried out by competent and experienced trainers<br>● Compliance with local regulations (experimental activities with animals must be carried out by personnel with the respective personal licenses)
**Recommendations:**<br>● Training activities should be regularly and independently reviewed / audited<br>● As far as possible, provide more than one person with the competencies to carry out a given procedure to limit operator effect<br>● Users must undergo refresher training if they have not done a technique for a period of time (e.g. 6 months) to ensure correct animal handling and to mitigate potential safety risks<br>● Training of applicable guidelines (e.g. PREPARE, FAIR, ARRIVE)<br>● Knowledge transfer by regular exchange of experience e.g. via meetings and dedicated phenotyping training workshops across centres in the context of INFRAFRONTIER and the IMPC
**References:**<br>● DIRECTIVE 2010/63/EU (EU Parliament and Council 2010) and its applicable national transitions<br>● ISO 9001 Quality Management Systems (ISO 2015)

- AAALAC Accreditation Program (AAALAC 2021)
- Handbook: Quality practices in basic biomedical research (WHO TDR 2006). Chapter 4.1.2 Personnel and training
- Trainings offered on INFRAFRONTIER website (INFRAFRONTIER 2021)

INFRAFRONTIER Quality Principle (6) in Systemic Phenotyping

(6) We apply reference ranges where feasible

Context:

- The reference range reflects the biological / technical variation of a parameter by analysing control groups and is specific to a laboratory and its procedures
- Reference ranges are important tools to ensure quality of data (monitoring over time, identification of technical problems, and biologically impossible outliers) and to identify systematic shifts
- A reference range is possible only with a large-scale project (e.g. n >40, depending on test)
- Reference ranges may also be used to identify potential phenotypes in experimental animals (see principle 8, reference range model)

Requirements:

- Reference ranges must be established on control mice using the same procedures, conditions, age, sex, and genetic background
- Control (if possible littermates) and experimental mice should be tested concurrently

Recommendations:

- Record and analyse the variability of a given parameter
- Record and analyse the stability of the variability
- Record and analyse the temporal stability of the mean
- Use accumulated wild-type data to identify and refine the reference range

References:

- Karp et al 2012
- Karp et al 2014
- NCCLS document C28-A2 (Clinical Laboratory and Standards Institute 2000)

INFRAFRONTIER Quality Principle (7) in Systemic Phenotyping

(7) We exchange and analyse reference lines or reference samples where feasible

Context:

- Test inter-centre reproducibility:
    - Compare the genotype effect across centres
    - Analyse the concordance for phenotyping tests across centres

Requirements:

- Use robust, validated phenotyping tests
- Compare data from control mice between centres
- Apply the same procedure at the same time, age, sex, background (common pipeline between centres)
- Collect and consider differences in the metadata (e.g. housing conditions, diet formulation and batch number)
- Analyse the inter-centre heterogeneity visually and by using meta-analytical measures (see reference)
- Perform regular re-evaluations, e.g. by analysis of current data, on a periodic basis (e.g. 5 years), and decide on the necessity to re-analyse the reference line
- Statistical power analysis *a priori* to determine the minimum cohort size required for the tests (consider sexual dimorphism or other co-variants like body weight)

Recommendations:

- Exchange the same mutant line or biological samples between centres, especially when new tests have been implemented
- When discordance between centres occurs, consider metadata differences and the possibility of false negative or positive results

References:

- de Angelis et al 2015

INFRAFRONTIER Quality Principle (8) in Systemic Phenotyping

(8) We use appropriate statistical analyses that are fit for purpose

Context:

- Statistical analysis is the process of generating statistics from stored data and analysing the results to deduce or infer meaning about the underlying dataset or the reality that it attempts to describe. In addition, it empowers to ensure reproducibility while minimising the number of animals required.
- Statistical analysis may be used to:
    - Present key findings revealed by a dataset
    - Summarise information
    - Calculate measures of cohesiveness, relevance, or diversity in data
    - Make future predictions based on previously recorded data
    - Test experimental predictions
- T-test, Wilcoxon-Mann-Whitney test, Fisher's exact test, reference range (plus) method, and linear mixed models are used to compare the experimental groups

Requirements:

- Statistical power analysis *a priori* to determine the minimum cohort size required for the tests and *a posteriori* to validate the hypothesis
- Statistical analysis should be performed and reported (e.g. test method, software used)

- Analyse based on the assay and the structure of the data
- Know the assumption and conditions of the statistical methods
- Know the type of the data collected and objective of the study

Recommendations:

- Work with biostatisticians to define the most appropriate analysis method
- Use current version of PhenStat tool (see Kurbatova et al 2015, 2020)
- Apply soft windowing for large datasets with running baseline (see Karp et al 2014)

References:

- ARRIVE guidelines (Kilkenny et al 2010, Percie du Sert 2020a, Percie du Sert 2020b)
- Haselimashhadi et al 2020
- Karp et al 2014
- Karp et al 2015
- Kurbatova et al 2015
- Mishra et al 2019
- White et al 2013
- PhenStat Tool (Kurbatova et al 2020)
- DataAnalytiks.org.uk. (2019): https://www.dataanalytics.org.uk/data-analytics-knowledge-base-tips-tricks-r-excel/statistics-guide/which-statistics-test/
- IMPC website (IMPC 2021): statistical-analysis (https://www.mousephenotype.org/help/data-analysis/statistical-analysis/), statistics-help (https://www.mousephenotype.org/data/documentation/statistics-help)

INFRAFRONTIER Quality Principle (9) in Systemic Phenotyping

(9) We report metadata

Context:

- Experimental Metadata is important for understanding and interpreting experimental data
- Metadata consists of additional parameters that the area experts have determined as important for capture, and that could explain potential variation in the experimental readouts of interest
- Metadata Parameter - a Metadata Parameter is not for collecting experimental values but provides information about the conditions the experiment was done under or about the equipment used (definition taken from IMPReSS / glossary, see https://www.mousephenotype.org/impress/glossary (IMPC 2021)). Equipment Manufacturer, Experimenter ID and Period of fasting are examples of Metadata Parameters. Some of these parameters can influence the measurements.

Requirements:

- Record the health status, housing and husbandry conditions and capture them for data analysis (e.g. diet, bedding, day-night cycle, air renewal)
- Record the operational conditions and capture them for data analysis (e.g. number of mice per cage, mutants and controls grouped or not?)
- Record the experimental conditions (e.g. fasting time and duration, which anaesthesia, route of blood collection, time of analysis)
- Record equipment information to be tracked (e.g. ID, Manufacturer, Model)
- Refer to IMPC list of experimental metadata:
  - IMPReSS (see IMPReSS 2021)
  - IMPC Pipeline Protocols include metadata for each test (example: https://www.mousephenotype.org/impress/ParameterInfo?action=list&procID=623, accessed 22 April 2021)

Recommendations:

- Metadata should be collected in a reproducible and standardised format
- Metadata should comply with FAIR data principles (Findable, Accessible, Interoperable, Reusable; see Quality Principle (10))
- Research data associated metadata should be provided (refer to ARRIVE and PREPARE guidelines)
- High standard in data and metadata management should be ensured

References:

- PREPARE guidelines (Smith et al 2018)
- ARRIVE guidelines (Kilkenny et al 2010, Percie du Sert 2020a, Percie du Sert 2020b)
- Blumzon et al 2019
- Karp et al 2015
- Riley et al 2017
- Wilkinson et al 2016
- IMPReSS website (IMPReSS 2021)
- IMPC website (IMPC 2021)

INFRAFRONTIER Quality Principle (10) in Systemic Phenotyping

(10) We advise that the data that we provide is FAIR (Findable, Accessible, Interoperable, Reusable)

Context:

- Meaning of FAIR (according to go-fair.org (GO FAIR 2021)):
  - Findable: metadata and data should be easy to find for both humans and computers
  - Accessible: knowledge about how data can be accessed, possibly including authentication and authorisation
  - Interoperable: data usually needs to be integrated with other data. In addition, the data needs to interoperate with applications or workflows for analysis, storage, and processing
  - Reusable: optimise the reuse of data. To achieve this, metadata and data should be well-described so that they can be replicated and/or combined in different settings

- FAIR data and services aim to improve reproducibility of biomedical research, to drive new scientific discoveries and to enable any researcher in Europe and across the world to access and use them (from eosc-life.eu (EOSC-Life 2021))

Requirements:

- Consider FAIR principles in each phase of study
- Ensure that personnel involved in data curation processes are trained in the meaning and application of FAIR principles

Recommendations:

- To be Findable (Wilkinson 2016)**:**
  - F1. (Meta)data are assigned a globally unique and persistent identifier
  - F2. Data are described with rich metadata (defined by R1 below)
  - F3. Metadata clearly and explicitly include the identifier of the data they describe
  - F4. (Meta)data are registered or indexed in a searchable resource
- To be Accessible (Wilkinson 2016)**:**
  - A1. (Meta)data are retrievable by their identifier using a standardised communications protocol
    - A1.1 The protocol is open, free, and universally implementable
    - A1.2 The protocol allows for an authentication and authorisation procedure, where necessary
  - A2. Metadata are accessible, even when the data are no longer available
- To be Interoperable (Wilkinson 2016)**:**
  - I1. (Meta)data use a formal, accessible, shared, and broadly applicable language for knowledge representation
  - I2. (Meta)data use vocabularies that follow FAIR principles
  - I3. (Meta)data include qualified references to other (meta)data
- To be Reusable (Wilkinson 2016):
  - R1. Meta(data) are richly described with a plurality of accurate and relevant attributes
    - R1.1. (Meta)data are released with a clear and accessible data usage licence
    - R1.2. (Meta)data are associated with detailed provenance
    - R1.3. (Meta)data meet domain-relevant community standards

References:

- Wilkinson et al 2016
- EOSC-Life website: https://www.eosc-life.eu (EOSC-Life 2021)
- GO FAIR website: www.go-fair.org (GO FAIR 2021)
- IMPC website: https://www.mousephenotype.org (IMPC 2021; IMPC data as example as example for FAIR data)

INFRAFRONTIER Quality Principle (11) in Systemic Phenotyping

(11) We maintain and extend the mechanisms (working groups, training, exchange of experience) to constantly improve our data quality (Plan-Do-Check-Act)

Context:

- PDCA (Plan-Do-Check-Act) is an iterative, four-stage approach for continually improving processes, products, or services, and for resolving problems. It involves systematically testing possible solutions, assessing the results, and implementing the ones proven to work.

  The PDCA Cycle provides a simple and effective approach for solving problems and managing change. It enables businesses to develop hypotheses about what needs to change, to test these hypotheses in a continuous feedback loop, and to gain valuable learning and knowledge.

  The PDCA cycle consists of four components:
  - *Plan:* Identify the problem, collect relevant data, understand the problem's root causes, and prioritise which ones to test
  - *Do*: Develop a potential solution, decide upon a measurement to gauge its effectiveness, and test the solution
  - *Check*: Confirm the results through pre- and post- data comparison, study the result, measure effectiveness, and determine whether the problem was resolved
  - *Act:* Document the results. If the solution was successful, implement it and inform others about process changes. If not successful, tackle the next possible cause and repeat the PDCA cycle again
- INFRAFRONTIER: to improve our processes we perform inter-centre exchange of experience on quality management and principles through working groups, also considering novel approaches and technologies as discussed at INFRAFRONTIER/IMPC phenotyping workshops and scientific meetings

Requirements:

- Commitment to quality policy and principles from all personnel at all levels
- Implement quality related processes that enable the consistency and effectiveness of the quality management to be checked against the set objectives, e.g.
  - Error management
  - Collection of stakeholder complaints and satisfaction surveys and definition and analysis of corresponding measures
  - Process monitoring by reviewing of key performance indicators
  - Assign an independent internal reviewer to assess processes and recommend improvements
  - Invest in training of quality awareness (also refer to Quality Principle (5))
- The outcome of quality related processes are corrective and/or preventive actions and action plans to support continuous improvement

Recommendations:

- Consider a common set of principles to ensure quality, such as Quality Management System (QMS) requirements stated in e.g. ISO, Good Laboratory Practice (GLP), AAALAC, and aim for certification/accreditation
- Consider establishing a Quality Manual / top-level document that describes an organisation's QMS
- Consider risk and opportunity management (e.g. ISO 9001)
- Consider regular quality reviews (e.g. annually)
- Consider participating in a (QM) network of institutions in similar scientific research areas as an opportunity to address common scientific quality problems, e.g. INFRAFRONTIER / IMPC working groups
- Consider external reviews or site visits by domain experts
- Consult OECD guidelines

References:

- AAALAC Accreditation Program (AAALAC 2021)
- ISO 9001 Quality Management Systems (ISO 2015)
- Principles on Good Laboratory Practice (GLP) (OECD 1998)
- Reference framework for assessing the impact of research infrastructures (OECD 2019)
- QM framework in BMS RIs (BBMRI-ERIC et al 2017)
- Ibisa website (IBiSA 2021): IQuaRe, French network of technological research platforms

List of References

AAALAC (2021) Association for Assessment and Accreditation of Laboratory Animal Care International https://www.aaalac.org. Accessed 19 April 2021

BBMRI-ERIC, EATRIS, ECRIN, ELIXIR, EMBRC, EMPHASIS et al. (2017) A framework for quality management in the biomedical research infrastructures (BMS RIs). http://doi.org/10.5281/zenodo.834333

Blumzon CFI, Pănescu AT (2019) Data Storage. In: Bespalov A, Michel M, Steckler T (eds) Good Research Practice in Non-Clinical Pharmacology and Biomedicine. Handbook of Experimental Pharmacology, vol 257. Springer, Cham, pp 277-297, https://doi.org/10.1007/978-3-030-33656-1

Canadian Council on Animal Care (CCAC) (2019). CCAC guidelines: Mice. https://www.ccac.ca/CCAC_Guidelines_Mice.pdf. Accessed 23 Apr 2021

Canadian Council on Animal Care (CCAC) (2020) Guide to the Care and Use of Experimental Animals. Vol 1, 2nd edition. https://www.ccac.ca/Documents/Standards/Guidelines/Experimental_Animals_Vol1.pdf. Accessed 23 Apr 2021

Clinical Laboratory and Standards Institute (2000) NCCLS. How to Define and Determine Reference Intervals in the Clinical Laboratory; Approved Guideline—Second Edition. NCCLS document C28-A2. NCCLS, Wayne.

DataAnalytiks.org.uk. (2019) Choosing the right statistical test/analysis. https://www.dataanalytics.org.uk/data-analytics-knowledge-base-tips-tricks-r-excel/statistics-guide/which-statistics-test/. Accessed 19 April 2021

de Angelis MH, Nicholson G, Selloum M et al (2015) Analysis of mammalian gene function through broad-based phenotypic screens across a consortium of mouse clinics. Nat Genet 47(9):969-978. https://doi.org/10.1038/ng.3360

EOSC-Life (2021) Building a digital space for the life sciences. https://www.eosc-life.eu. Accessed 19 April 2021

EU Parliament and Council (2010) DIRECTIVE 2010/63/EU OF THE EUROPEAN PARLIAMENT AND OF THE COUNCIL on the protection of animals used for scientific purposes, and applicable national legislation. Official Journal of the European Union L 276/33-79, 20.10.2010. https://eur-lex.europa.eu/DIRECTIVE 2010/63/EU. Accessed 23 Apr 2021

European Commission (2020) COMMISSION IMPLEMENTING DECISION (EU) 2020/569 establishing a common format and information content for the submission of the information to be reported by Member States pursuant to Directive 2010/63/EU of the European Parliament and of the Council on the protection of animals used for scientific purposes and repealing. Commission Implementing Decision 2012/707/EU. Official Journal of the European Union L 129/16 -50, 24.4.2020. https://eur-lex.europa.eu/COMMISSION IMPLEMENTING DECISION (EU) 2020/569. Accessed 23 Apr 2021

European Commission Expert Working Group (2012) National Competent Authorities for the implementation of Directive 2010/63/EU on the protection of animals used for scientific purposes. Working document on a severity assessment framework. https://ec.europa.eu/environment/chemicals/lab_animals/pdf/Endorsed_Severity_Assessment.pdf. Accessed 23 Apr 2021

European Commission Expert Working Group (2013) National Competent Authorities for the implementation of Directive 2010/63/EU on the protection of animals used for scientific purposes. CORRIGENDUM of 24 January 2013. Working document on genetically altered animals. https://ec.europa.eu/EWG/genetically_altered_animal_corrigendum.pdf. Accessed 23 Apr 2021

European Commission Expert Working Group (2014) National Competent Authorities for the implementation of Directive 2010/63/EU on the protection of animals used for scientific purposes. A working document on Animal Welfare Bodies and National Committees to fulfil the requirements under the Directive. https://ec.europa.eu/environment/chemicals/lab_animals/pdf/endorsed_awb-nc.pdf. Accessed 23 Apr 2021

European Commission, Joint Research Centre (2019) EURL ECVAM dataset on alternative methods to animal experimentation (DB-ALM). http://data.europa.eu/89h/b7597ada-148d-4560-9079-ab0a5539cad3. Accessed 22 April 2021

European Medicines Agency (EMA) (2016) Guideline on the principles of regulatory acceptance of 3Rs (replacement, reduction, refinement) testing approaches. EMA/CHMP/CVMP/JEG-3Rs/450091/2012. https://www.ema.europa.eu/guideline-principles-regulatory-acceptance-3rs-replacement-reduction-refinement-testing-approaches_en.pdf. Accessed 23 Apr 2021

GO FAIR (2021) www.go-fair.org. Accessed 19 April 2021

Haselimashhadi H, Mason JC, Munoz-Fuentes V, et al (2020) Soft windowing application to improve analysis of high-throughput phenotyping data. Bioinformatics 36(5):1492-1500. https://doi.org/10.1093/bioinformatics/btz744

IBiSA (2021) IQuaRe. https://www.ibisa.net/la-mission-qualite.php#bloc-2-. Accessed 19 April 2021

IMPC (2021) International Mouse Phenotyping Consortium. https://www.mousephenotype.org. Accessed 19 April 2021

IMPReSS (2021) International Mouse Phenotyping Resource of Standardised Screens. https://www.mousephenotype.org/impress. Accessed 19 April 2021

INFRAFRONTIER (2021) mouse disease models. https://www.infrafrontier.eu. Accessed 19 April 2021

International Organization for Standardization (ISO) (2015) ISO 9001:2015 Quality management systems — Requirements. https://www.iso.org/obp/ui/#iso:std:iso:9001:ed-5:v1:en. Accessed 23 Apr 2021

Karp NA, Melvin D, Sanger Mouse Genetics Project, Mott RF (2012) Robust and Sensitive Analysis of Mouse Knockout Phenotypes. PLoS ONE 7(12): e52410. https://doi.org/10.1371/journal.pone.0052410

Karp NA, Speak AO, White JK, Adams DJ, Hrabe ́ de Angelis M et al (2014) Impact of Temporal Variation on Design and Analysis of Mouse Knockout Phenotyping Studies. PLoS ONE 9(10): e111239. https://doi.org/10.1371/journal.pone.0111239

Karp NA, Meehan TF, Morgan H, Mason JC, Blake A, Kurbatova N et al (2015) Applying the ARRIVE Guidelines to an In Vivo Database. PLoS Biol 13(5): e1002151. https://doi.org/10.1371/journal.pbio.1002151

Kilkenny C, Browne WJ, Cuthill IC, Emerson M, Altman DG (2010) Improving Bioscience Research Reporting: The ARRIVE Guidelines for Reporting Animal Research. PLoS Biol 8(6): e1000412. https://doi.org/10.1371/journal.pbio.1000412

Kurbatova N, Mason JC, Morgan H, Meehan TF, Karp NA (2015) PhenStat: A Tool Kit for Standardized Analysis of High Throughput Phenotypic Data. PLoS ONE 10(7): e0131274. https://doi.org/10.1371/journal.pone.0131274

Kurbatova N, Karp N, Mason J, Haselimashhadi H (2020) PhenStat: Statistical analysis of phenotypic data. R package version 2.26.0. https://doi.org/doi:10.18129/B9.bioc.PhenStat

Meehan TF, Conte N, West DB et al (2017). Disease model discovery from 3,328 gene knockouts by The International Mouse Phenotyping Consortium. Nat Genet 49(8):1231-1238. https://doi.org/10.1038/ng.3901

Mishra P, Pandey CM, Singh U, Keshri A, Sabaretnam M (2019) Selection of appropriate statistical methods for data analysis. Ann Card Anaesth. 22(3):297-301. https://doi.org/10.4103/aca.ACA_248_18

National Research Council (2011) Guide for the Care and Use of Laboratory Animals: Eighth Edition. The National Academies Press, Washington DC. https://doi.org/10.17226/12910

NC3Rs (2021) UK National Centre for the Replacement, Refinement and Reduction in Animals in Research. https://www.nc3rs.org.uk. Accessed 19 April 2021

NIH (2021) National Institutes of Health. https://www.nih.gov. Accessed 19 April 2021

Organization for Economic Co-operation and Development (OECD) (1998) Series on Principles of Good Laboratory Practice and Compliance Monitoring. No. 1: OECD Principles on Good Laboratory Practice. ENV/MC/CHEM(98)17. http://www.oecd.org/officialdocuments/publicdisplaydocumentpdf/?cote=env/mc/chem(98)17&doclanguage=en. Accessed 23 Apr 2021

Organization for Economic Co-operation and Development (OECD) (2019) Reference framework for assessing the scientific and socio-economic impact of research infrastructures. OECD Science, Technology and Industry Policy Papers No. 65. OECD Publishing, Paris. https://doi.org/10.1787/3ffee43b-en

Percie du Sert N, Ahluwalia A, Alam S, Avey MT, Baker M, Browne WJ et al. (2020a) Reporting animal research: Explanation and elaboration for the ARRIVE guidelines 2.0. PLoS Biol 18(7): e3000411. https://doi.org/10.1371/journal.pbio.3000411

Percie du Sert N, Hurst V, Ahluwalia A, Alam S, Avey MT, Baker M et al (2020b) The ARRIVE guidelines 2.0: Updated guidelines for reporting animal research. PLoS Biol 18(7): e3000410. https://doi.org/10.1371/journal.pbio.3000410

Riley J (2017) Understanding metadata – what is metadata, and what is it for? National Information Standards Organization (NISO), Baltimore. Available via NISO. https://www.niso.org/publications/understanding-metadata-2017. Accessed 23 Apr 2021

Smith AJ, Clutton RE, Lilley E, Aa Hansen KE, Brattelid T (2018) PREPARE: guidelines for planning animal research and testing. Lab Anim 52(2): 135–141. https://dx.doi.org/10.1177%2F0023677217724823

UK Statutory Instruments (2012) The Animals (Scientific Procedures) Act 1986 Amendment Regulations 2012. SI 4 2012/3039. https://www.legislation.gov.uk/uksi/2012/3039/contents/made. Accessed 23 Apr 2021

White JK, Gerdin AK, Karp NA et al (2013) Genome-wide generation and systematic phenotyping of knockout mice reveals new roles for many genes. Cell 154(2):452-64. https://doi.org/10.1016/j.cell.2013.06.022

WHO Special Programme for Research and Training in Tropical Diseases (TDR) (2006) Handbook: Quality practices in basic biomedical research. WHO, Geneva. Available at https://www.who.int/tdr/publications/training-guideline-publications/handbook-quality-practices-biomedical-research/en/. Accessed 23 Apr 2021

Wilkinson M, Dumontier M, Aalbersberg I et al (2016) The FAIR Guiding Principles for scientific data management and stewardship. Sci Data 3, 160018. https://doi.org/10.1038/sdata.2016.18
